# Generation of selenium nano- and micro-materials using abiotic and biotic processes

**DOI:** 10.46439/signaling.4.101

**Published:** 2026

**Authors:** Michael Wells, Kyle Rugg, Humaira Urmee, Jenna Etheridge, Caitlyn Boudreaux, Natalie Fairley, Mark A. DeCoster

**Affiliations:** 1 Biological Sciences, Louisiana Tech University, USA; 2 Cellular Neuroscience Lab, Biomedical Engineering, Louisiana Tech University, USA; 3 Institute for Micromanufacturing, Louisiana Tech University, USA

**Keywords:** Selenium, Cancer, Nanomaterials, Copper, Microbes, Metal-Organic Biohybrids (MOBs)

## Abstract

We have previously described the generation of Metal Organic Biohybrids (MOBs) as self-assembling structures that are formed by a combination of a biological component (amino acid), and a metal, such as copper. This MOBs synthesis process has now been distilled to be purely biochemical (a green chemistry process), or abiotic, not requiring the presence of cells. Building on these discoveries, in this commentary we describe the use of selenium for the first time using MOBs synthesis techniques, as well as using biotic techniques, that is, selenium in the presence of living microbes. New and different materials are described using the respective techniques, which have different nano- and micro-scale properties. We anticipate that the abiotic and biotic techniques may provide parallel methods for generating new MOBs and new nanomaterials, respectively. One potential application of the generated materials could include treatments for cancer, since in the case of selenium-containing MOBs, we demonstrate 100 percent biodegradability.

## Commentary

Metal Organic Biohybrids (MOBs) are novel micro- and nanomaterials that have been described by us as self-assembling structures comprised of a biological component (amino acid), and a metal, such as copper. The first MOB that we discovered and described used cystine as the amino acid and copper sulfate pentahydrate as the copper component to form copper high-aspect ratio structures or CuHARS [1-3]. Since CuHARS, we have also described MOBs using silver, which form nanoparticles (AgCysNPs), and MOBs using cobalt (CoMOBs) [3,4]. Here for the first time, we also describe MOBs generated using selenium in the form of selenocystine, which we name as SeMOBs, and in parallel, selenium nanoparticles generated using microbes. Although not tested directly here, these materials could have anti-cancer potential, for example through mechanisms such as apoptosis and cuproptosis. The work presented here highlights conceptual advances and early observations rather than full mechanistic characterization.

The generation of MOBs is a 100% green chemistry (biochemical) and abiotic process, meaning all processes are carried out at body temperature (37°C) or lower. Thus, MOBs synthesis could be performed at an industrial scale, and the use of amino acid portions indicates that MOBs could have substantial untapped biomedical utility. Crucially, while the four different MOBs we have discovered are all self-assembled and formed under biological conditions, they do not require the presence of cells to form, although the original discovery of CuHARS did occur in the presence of cells [1]. Thus, MOBs can be generated abiotically.

Our aim in this commentary is to provide preliminary evidence for the potential applicability of MOBs in biomedical research. The first evidence comes from an area of biological research seldom considered in biotechnology, that of the origin of life. Thus, a biotic process, involving microbes, may be considered for generating metal-derived materials as well. The essence of origin of life research is to understand how a wet, rocky world composed of geochemical reactions, gave rise to biochemical reactions [5] that sustain and co-evolve with biogeochemical cycles of elements in the periodic table [6,7]. Of especial interest is the anaerobic Wood-Ljungdahl pathway, which is the only pathway for carbon fixation in organisms which generates energy (i.e., ATP production) from CO_2_ reduction. It was likely present in the last universal common ancestor of all extant cellular lineages [8,9]. Organic carbon byproducts of the Wood-Ljungdhal pathway are identical to many of the abiotically generated organic carbon molecules, including formate and acetate, found in alkaline hydrothermal vent systems [10] on the modern Earth. Alkaline hydrothermal vent fields are the only environments on Earth known to spontaneously mimic chemiosmotic mechanisms (i.e., energy production coupled to carbon fixation from collapsing pH gradients) and thus are of great interest to origin of life research [5,9].

Key enzymes in the Wood-Ljungdahl pathway are metalloenzymes that harbor nickel, iron, and molybdenum/tungsten co-factors. These include nickel iron (selenium) hydrogenases [11], molybdopterin/tungstopterin guanine dinucleotide co-factor binding members of the MopB superfamily [12], and enzymes that require [4Fe-4S] or [2Fe-2S] co-factors such as ferredoxins [9,13]. Often these critical co-factors are coordinated to the enzymes via cysteine or selenocysteine, molecules we have used previously, or here, for MOBs synthesis. The only exceptions are evolutionarily derived members of the MopB superfamily which can exploit serine or aspartate residues for co-factor coordination [12]. The shapes of these co-factors are reminiscent of common minerals found in alkaline hydrothermal vent pores [14]. Nanocrystals of these minerals, such as greigite (Fe_3_S_4_), magnetite (Fe_3_O_4_), and awaruite (Ni_3_Fe), by themselves can couple CO_2_ reduction to H_2_ oxidation and generate mM and μM quantities of key organic acids including formate, acetate, and pyruvate [15]. In other words, the minerals of these vent pores are themselves capable of carrying out biochemical reactions without need of enzymes, much less cells.

It is remarkable what emerges when dissolved ferric chloride and sodium sulfide are exposed to μM cysteine in synthetic media mimicking anoxic alkaline hydrothermal vent fluids [16]. These experimental conditions generated many [2Fe-2S] and [4Fe-4S] clusters whose structural geometry is highly similar to the structure of the iron-sulfur cluster co-factors of key enzymes, including ferredoxin and membrane-bound hydrogenases, in the acetogenic pathway.

It occurred to us that the abiotically established MOBs synthesis process could be compared to biologically generated nanoparticles by culturing anaerobic bacteria with metals or metalloids exploited in successful MOBs syntheses, to test the hypothesis that biomedically promising novel nanoparticles and nano- and micro-materials could be generated via the MOBs synthesis. Copper is not a practical choice given the profound toxicity of copper to anaerobes [17]. Bacterial reduction of sulfur oxyanions, while widespread, does not result in the formation of sulfur nanoparticles. However, reductive transformations of selenium oxyanions does result in elemental selenium nanospheres [18].

Selenium nanoparticles have been shown to exhibit anticancer activity, and two of the hypothesized mechanisms of cancer cytotoxicity include stimulation of cell signaling pathways for apoptosis [19-21] and autophagy [22,23]. While SeNP provision has been shown to stimulate a bevy of pro-apoptotic pathways [20], the Beclin-1 cell signaling pathway appears to be a key mechanism that is common for both observed apoptosis and autophagy in studied cancer cell lines [20,23]. Both pathways are stimulated in cancer cell lines by the increased expression of selenoproteins in cancer cells caused by doses of SeNPs [21]. This led us to wonder if selenocystine could replace cystine in MOBs synthesis and enhance the cytotoxicity of CuHARS by similarly enhancing the expression of selenoproteins in cancer cell lines. We provide here some initial observations exploiting selenium materials with both methods: abiotic assembly to form selenium-containing MOBs, and biotic processes to form pure selenium nanoparticles.

**Figure 1.**
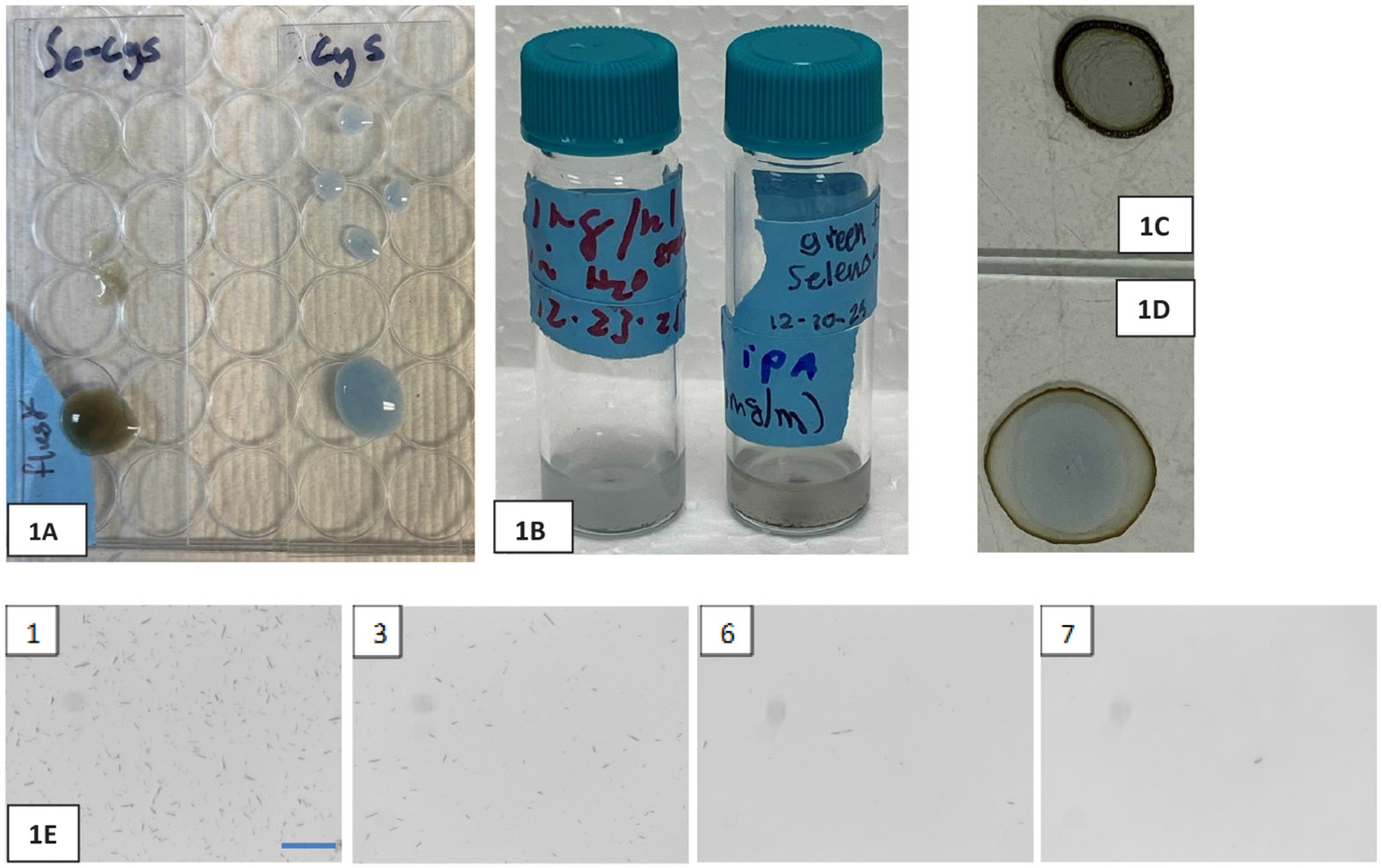
**Panel 1A.** SeMOBs generated with selenocystine (left slide) or CuHARS generated with cystine (right slide), were spotted onto glass slides and imaged for comparative color differences. **Panel 1B.** Stability of SeMOBs in water (left vial) or 70% isopropanol (right vial). **Panel 1C.** The colloidal supernatant generated from SeMOBs demonstrated a dark coffee ring when dried on glass; in contrast, the SeMOBs pellet (**1D**) exhibited a thinner dark coffee ring with a characteristic blue color center suggestive of copper. **Panel 1E**. SeMOBs incubated in CRL2303 brain tumor cell media at 37°C in a CO_2_ incubator and tracked daily by monochrome camera brightfield microscopy. Breakdown is shown from left to right at 1, 3, 6, and 7 days *in vitro*; scale bar in 1st panel = 100 μm.

We found that selenocystine could be used in lieu of cystine for MOBs synthesis, and this resulted in materials with similar properties to CuHARS. SeMOBs were generated using previously published methods [1,3,4,24], but replacing the amino acid cystine with selenocystine. The metal component copper sulfate pentahydrate was then used to generate SeMOBs. A key difference between SeMOBs and previously reported MOBs is that, the abiotic process using selenocystine, resulted in two distinct materials components over a 5 hour period: 1) a copper-associated high-aspect ratio structure reminiscent of the CuHARS, which had a blue color, and 2) a brown-amber color material which was nanoparticulate in form and not elongated. These two distinct phases could be separated by centrifugation (Figure 1A). Like the CuHARS, SeMOBs themselves are remarkably stable once synthesized, dried, and then reconstituted in sterile water or isopropanol (Figure 1B), at a concentration of 1mg/ml. The SeMOBs demonstrated intense coffee ring formation [4,25]. When the colloidal supernatant was dried on glass, it had a uniform dark color and thick coffee ring (Figure 1C). In contrast, the settled pellet was mixed with dark particulates forming the coffee ring and the copper-containing elongated structures remaining in the center of the dried droplet with a characteristic blue color (Figure 1D).

We then compared the biodegradation potential of these materials to CuHARS, which had previously been shown to break down completely, albeit slowly, in cell culture media [24,26]. It was found that SeMOBs, like CuHARS, can completely be degraded in cell culture media, indicating their potential for drug delivery systems or to modify cell growth and morphology directly, as has been suggested for CuHARS [27,28]. In preliminary studies, we found that SeMOBs completely degraded within 7 days, in contrast to CuHARS, which took up to 18 days (Figure 1E) [26]. In parallel studies carried out at the same time, lower concentrations of SeMOBs broke down even faster (Figure 1E), further supporting the hypothesis that SeMOBs degradation under these conditions is faster than for CuHARS breakdown. Further experiments providing kinetic analysis, and replicate experiments for statistical analysis, are ongoing.

**Figure 2.**
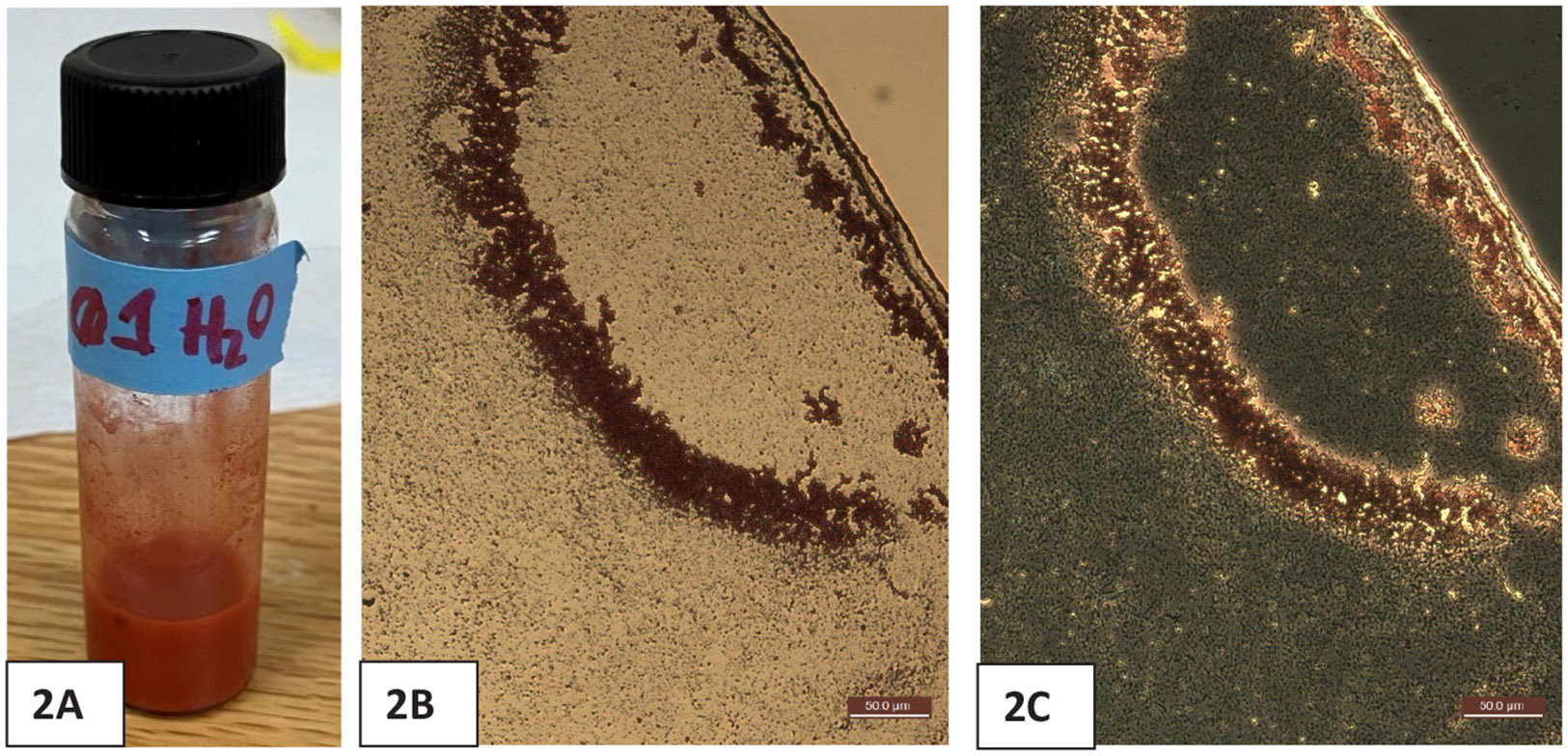
Seleno-nanosphere production from *Desulfitobacterium hafniense* PCP-1. Bulk production of the red product stably transferred to water (**2A**). Brightfield (**2B**) and phase (**2C**) microscopy of the red seleno-nanospheres. A droplet of material was dried on a glass slide, demonstrating coffee ring formation (with edge of ring in the upper right corner of 2B and 2C). Scale bar for both images= 50 μm.

For the biotic production of Se nanomaterials, the selenium respiring bacterium *Desulfitobacterium hafniense* PCP-1 was utilized. A wide array of bacteria and some archaea can conserve energy by exploiting the toxic, soluble selenium oxyanions selenate (Se(VI)) and selenite (Se(IV)) as terminal electron acceptors during anaerobic respiration [18]. This process typically results in red, amorphous, and non-toxic elemental selenium nanospheres with a diameter of roughly 200 to 250 nm [29]. *D. hafniense* PCP-1 was chosen as the chassis for selenium nanosphere production because it is one of the few strains of *D. hafniense* confirmed to respire Se(VI) and generate colloidal red selenium nanoparticles [30], and because PCP-1, like all other members of the *Desulfitobacterium* genus, is capable of detoxifying a staggering array of toxic metals, metalloids, and organohalides during anaerobic respiration [31]. This makes members of this genus highly desirable for bioremediation applications. The organism was cultivated with 20 mM sodium lactate as electron donor and 20 mM sodium selenate as terminal electron acceptor over 72 hours at 37°C. In contrast to the abiotic SeMOBs process, no copper was used in this biotic synthesis procedure. During this time, a vividly red colloidal form of elemental selenium was formed (Figure 2A).

Pristine SeNPs were formed using previously published methods [29]. When examined under the microscope, the generated material formed clear coffee-ring pattern when dried on glass (Figure 2B). As we observed with SeMOBs (See Figure 1B), Se nanoparticles which were formed biotically, were also highly stable once dried, weighed, and reconstituted in sterile water (Figure 2A).

**Figure 3.**
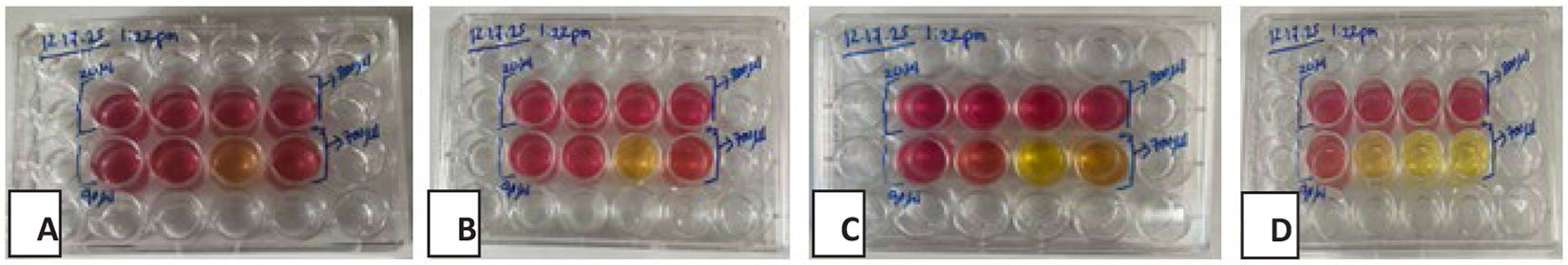
Cell growth in CRL2303 brain tumor cells over two weeks. Small, individual brain tumor cell spheroids were transferred to a 24 well plate and grown for 13 days, without changing media. Variation in growth rates and cell microenvironments was tracked daily, by changes in pH (phenol red indicator) with red indicating low growth (neutral pH), and yellow indicating high growth (acidic pH). From left to right (**Panels A-D**), cells were imaged at 1, 2, 8, and 13 days *in vitro*, respectively.

Now that these production systems have been constructed, we are interested in exploring their potential biomedical applications. Specifically, we hypothesize that these newly generated materials could have potential to kill or stress cancer cells by perturbing or activating cell signaling pathways (e.g. stimulate apoptosis). We will initiate these tests using a new model developed with the rapidly growing CRL2303 brain tumor cell line from ATCC [3,32], and applying our established cell spheroid (3 dimensional) cell model [33]. These cells in culture vary in their ability to rapidly proliferate, and metabolize the media, acidifying the environment, as indicated by intense color changes in the phenol red indicator commonly used in cell culture methods (Figure 3).

**Figure 4.**
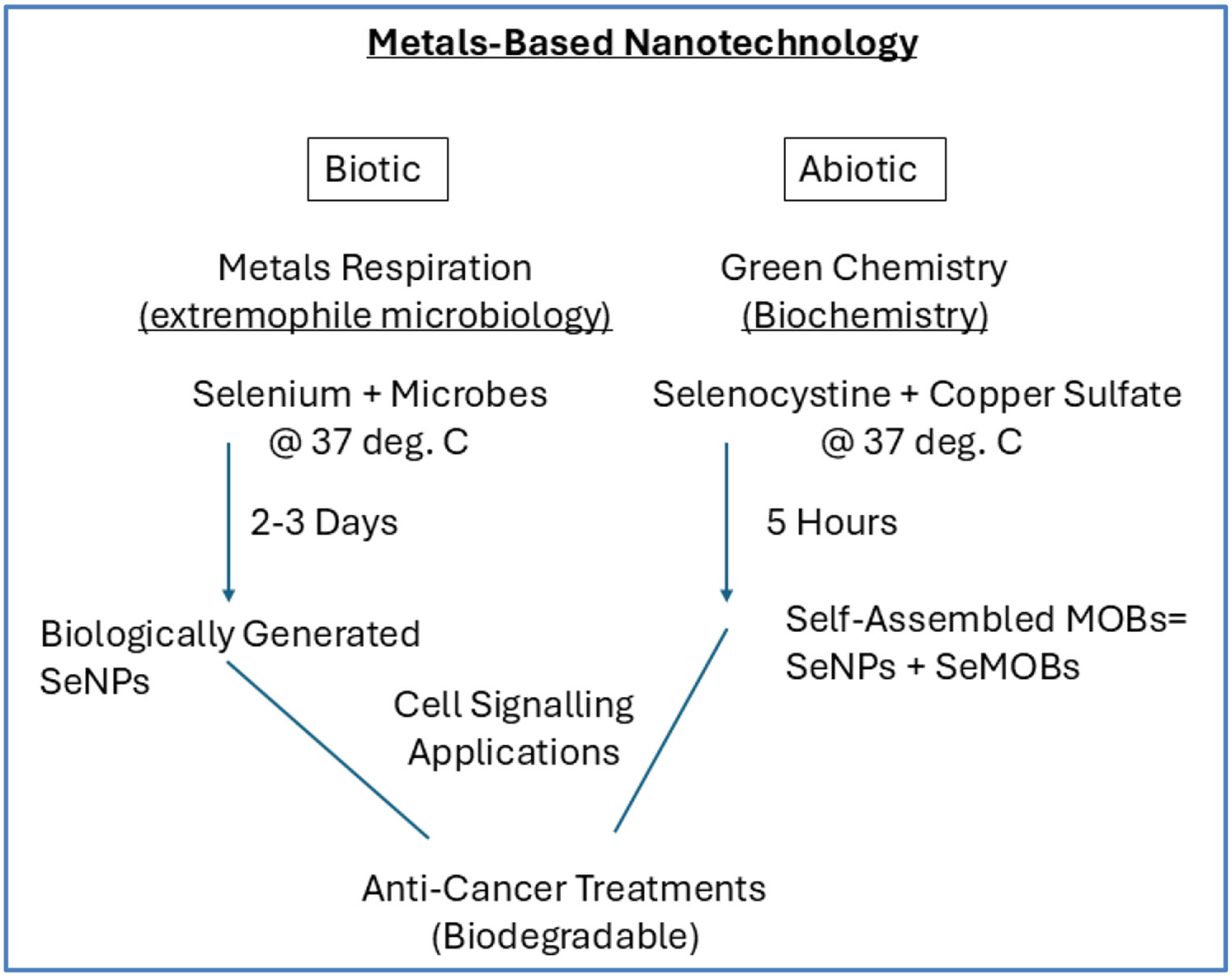
Schematic view of Biotic and Abiotic production of selenium-based nano- and micro-materials. Metals-based nanotechnology is being considered by using extremophile bacteria and archaea (the biotic route) that respire metals and metalloids, and the abiotic route (a biochemical route), which permits self-assembly of metals and amino acids to form metal-organic biohybrids (MOBs). Here, the material selenium was used as indicated in both environments to generate nano- and micro-materials of potential interest. We hypothesize that the SeMOBs generated abiotically may include both nanoparticles (SeNPs), and more elongated structures that have nano- and micro-features.

These changes in our model can be tracked over many days, since we use a cell-limiting method to start each well with only a few cells. We hypothesize that using this model, both biotically and abiotically generated metals-based nanomaterials can be tested for potential anti-cancer effects for further development (Figure 4, schematic). These observations of materials generation followed by biodegradation suggest that selenium-based MOBs and microbial selenium nanoparticles may warrant further investigation as biodegradable materials capable of interacting with cellular systems.

While both copper and selenium are used by normal cells and the body, excessive concentrations may be toxic and provide strategies for killing cancer cells [20,34]. From a signaling standpoint, potential mechanisms of cell killing could therefore include through endogenous pathways such as apoptosis and cuproptosis, with more recent signaling details becoming available for cuproptosis [35,36]. Both apoptosis and cuproptosis have been noted in potential signaling pathways involved in cancer cell killing, including brain tumor (glioma) cells [37-39]. While we have noted preliminary killing effects of our novel materials on glioma cells, we have not yet tested directly the potential cell signaling pathways involved. We might expect also that the selenium-containing materials generated biotically and abiotically could have different time-course and potency of potential anti-cancer effects, reflecting different cell signaling mechanisms, although we have not tested this directly. For example, the SeMOBs generated abiotically contain both copper and selenium as materials inputs during the synthesis process, while the SeNPs generated biotically lack any copper input during synthesis. Current studies are underway to further quantify the biophysical and dynamic properties of biotically and abiotically generated materials reported here. This will include the cytotoxicity of biotically synthesized SeNPs and SeMOBs, and elucidation of the structure and oxidation states of the copper and selenium embedded in these generated materials. For example, structural, spectroscopic, and chemical analyses including scanning electron microscopy with elemental analysis and Attenuated Total Reflectance Fourier-Transform Infrared (ATR-FTIR), are ongoing to distinguish input vs. output materials generated by the two respective abiotic and biotic process introduced here.
